# Supplementary material for: Active rheumatoid arthritis in a mouse model is not an independent risk factor for periprosthetic joint infection
Source: PLoS One. 2021 Aug 16;16(8):e0250910. doi: 10.1371/journal.pone.0250910 (PMC8366981; doi:10.1371/journal.pone.0250910)
Supplement: Attachment — Submitted filename: PONE-D-21-09243_reviewer.docx [file pone.0250910.s002.docx]

The submission from Rishi Trikha et al. reports that active RA in a mouse model is not an independent Risk Factor for Periprosthetic Joint Infection. The study is interesting and well structured. However, there are some corrections.

***Minor comments:***

1. ***Please refer to doi: 10.1016/j.biopha.2020.110023; 10.3390/app10041324***
2. ***The authors should include in the materials and methods section whether the mice used were male or female.***
3. ***The authors should better highlight the purpose of the study and the novelty***
4. ***The authors should better check the manuscript for any typographical errors.***
