# Supplementary material for: Active rheumatoid arthritis in a mouse model is not an independent risk factor for periprosthetic joint infection
Source: PLoS One. 2021 Aug 16;16(8):e0250910. doi: 10.1371/journal.pone.0250910 (PMC8366981; doi:10.1371/journal.pone.0250910)
Supplement: Attachment — Submitted filename: Response Letter PLOS ONE_RA.docx [file pone.0250910.s003.docx]

Dear Editorial Team at the Public Library of Science,

Thank you for taking the time to offer your suggestions. We truly appreciate your input and will address any/all concerns. Please see our responses to each reviewer below. All changes are line referenced.

**Reviewer #1:**

**This study investigated the mechanism underlining periprosthetic joint infection in active rheumatoid arthritis. The rationale behind the experiment was clear and straight forward. The manuscript is almost well written**

**While many different sources are used to set up the study in the introduction, little previous evidence is stated. The introduction is thus short and poorly sets up the rationale for the study. More attention to how this study fits into previous work in rheumatoid arthritis and inflammation should be added to improve this section. Please refer to doi: 10.3390/antiox9060511, 10.1186/s13075-019-2048-y. There are some minor grammar issues that should be fixed in order to aid the accessibility of the results to
the reader.**

Thank you for these astute comments. In an effort to better elucidate the rationale as well as the novelty of our study we have expanded the introduction and added to the discussion.

Lines 74-79, 115-117, 122-23 have been added to expand upon the importance of this study as well as the prevention of PJI in general.

Lines 81-84 have been reorganized and replaced at the beginning of the second paragraph of the introduction to ensure that the rationale of our study is better understood.

The authors have added text regarding the molecular basis of rheumatoid arthritis as well as novel therapeutics countering synovial inflammation referenced from both of the intriguing papers suggested (Lines 87-109).

The authors have further expanded on the molecular basis of collagen-induced arthritis (Lines 126-128).

The authors believe that the concluding sentence of the introduction now better highlights the rationale of our study (Lines 160-162).

The authors have added to and restructured the end of the discussion to ensure that the novelty, significance and rationale of the study are clearer (Lines 437-443).

The authors have made grammatical corrections to ensure accessibility.

**Reviewer #2: The submission from Rishi Trikha et al. reports that active RA in a mouse model is not an independent Risk Factor for Periprosthetic Joint Infection. The study is interesting and well structured. However, there are some corrections.**

**Minor comments:**

**1. Please refer to doi: 10.1016/j.biopha.2020.110023; 10.3390/app10041324**

**2. The authors should include in the materials and methods section whether the mice used were male or female.**

**3. The authors should better highlight the purpose of the study and the novelty**

**4. The authors should better check the manuscript for any typographical errors.**

1/3. Thank you for these insightful comments, we have referenced the article by Impellizzeri et al. to further explain the molecular basis of rheumatoid arthritis (Lines 87-88) as well as expand upon the molecular basis of collagen-induced arthritis (Lines 125-128). Furthermore, we have explained that while novel therapeutics are being studies (as referenced by Sunzini et al. along with two other studies), little evidence regarding the infectious burden of rheumatoid arthritis exists (Lines 107-108).

The authors believe that the purpose of this study has been set-up in a more coherent fashion by inserting/replacing the first sentence of the second paragraph of the introduction (Lines 81-84). The authors also believe that the novelty and significance of this study has been made clearer by incorporating the last sentence of the introduction (Lines 160-162) as well as the last three sentences of the discussion (Lines 437-443).

2. The authors have clarified that the mice used were male (Line 210); thank you for pointing this out.

4. The authors have made grammatical corrections to ensure accessibility.
